# Humans use Optokinetic Eye Movements to Track Waypoints for Steering

**DOI:** 10.1038/s41598-020-60531-3

**Published:** 2020-03-06

**Authors:** Otto Lappi, Jami Pekkanen, Paavo Rinkkala, Samuel Tuhkanen, Ari Tuononen, Juho-Pekka Virtanen

**Affiliations:** 1 Cognitive Science, Department of Digital Humanities & Helsinki Centre for Digital Humanities (Heldig), University of Helsinki, Helsinki, Finland; 2 TRUlab, University of Helsinki, Helsinki, Finland; 3 Spatial Planning and Transportation Engineering, Department of Built Environment, Aalto University, Espoo, Finland; 4 Department of Mechanical Engineering, Aalto University, Espoo, Finland; 5 Aalto University School of Engineering, Espoo, Finland

**Keywords:** Saccades, Smooth pursuit, Human behaviour

## Abstract

It is well-established how visual stimuli and self-motion in laboratory conditions reliably elicit retinal-image-stabilizing compensatory eye movements (CEM). Their organization and roles in natural-task gaze strategies is much less understood: are CEM applied in active sampling of visual information in human locomotion in the wild? If so, how? And what are the implications for guidance? Here, we directly compare gaze behavior in the real world (driving a car) and a fixed base simulation steering task. A strong and quantifiable correspondence between self-rotation and CEM counter-rotation is found across a range of speeds. This gaze behavior is “optokinetic”, i.e. optic flow is a sufficient stimulus to spontaneously elicit it in naïve subjects and vestibular stimulation or stereopsis are not critical. Theoretically, the observed nystagmus behavior is consistent with tracking waypoints on the future path, and predicted by waypoint models of locomotor control - but inconsistent with travel point models, such as the popular tangent point model.

## Introduction

Consider traveling in a textured environment at very high speed–for example rushing down a race track or an autobahn at 120+ mph; sledding down an olympic luge chute; a crazy head-first plummet in a bungee jump. The visual field a blur, eyes and concentration totally focused on where you are going. But at moderate speeds–the more sedate pace in which our sensory physiology evolved and where most of our lives are still spent - our experience is of a sharp, “unblurred”, and visually stable world. What is the basis of this visual stability?

There are central mechanisms for visual stability, such as saccadic suppression^1,2^ and remapping of receptive fields^3^, but active gaze strategies structuring the retinal input can also play a part. Our oculomotor system has a suite of reflexes and action patterns such as optokinetic response (OKR), vestibulo-ocular response (VOR), smooth pursuit and vergence that potentially could be used to stabilize the retinal image - and thus directly contribute to the experience of stability and focus. Indeed, in the laboratory studies of slow eye movements (SEM), it is commonly taken as given - if not definitional - that “in the wild” the job of these mechanisms is to prevent the retinal image from blurring and keep (relevant portions of) the visual world sharp and in focus. For reviews on these “compensatory eye movements” (CEM) see^4–7^.

We investigated the presence and parametric behavior of CEM in real and simulated high-speed locomotion (steering a bend). The theoretical rationale is that different visual steering models in the literature assume different gaze strategies. A travel-point strategy means the you hold gaze fixed, relative to the locomotor reference frame of the torso (or vehicle). Gaze lands on a point, say 20 m ahead on the path of travel, which travels along with you, so that the point your gaze lands on sweeps along the road as you move. A waypoint strategy, on the other hand, means the you fixate a point on the road, a specific location in the world you are going to pass over. In egocentric coordinates the fixation point therefore moves relative to you, as you approach the fixed location. Which strategy is being used can be differentiated by measuring CEM, as only the waypoint strategy implies CEM, which, moreover should scale with observer motion in predictable ways^8^.

**Figure 1:**
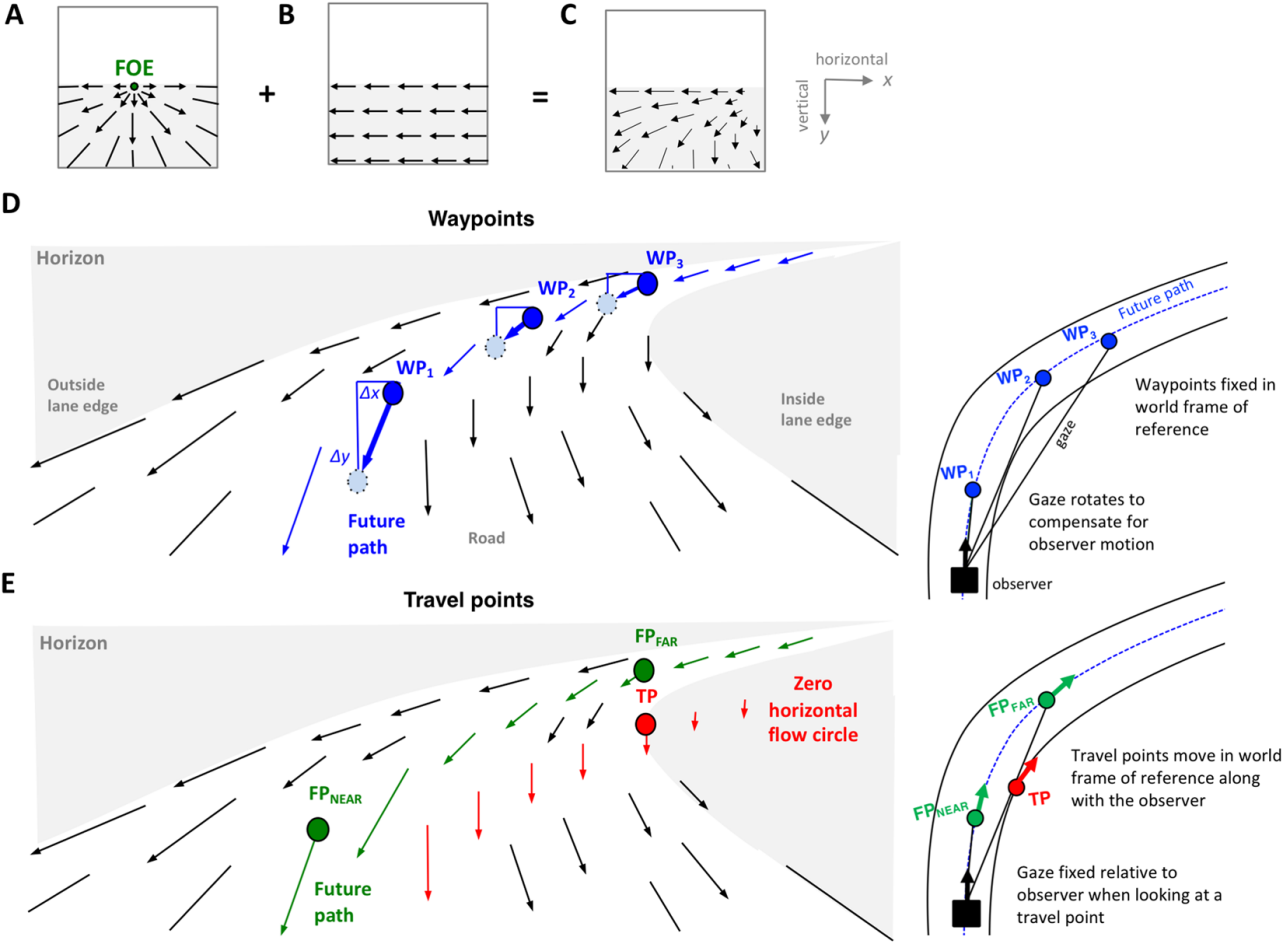
Schematic illustration of the egocentric visual flow field generated by different types of observer motion (**A**–**C**), with different steering points posited in the literature (**D**,**E**). (**A**) During linear translation on a plane, radial visual flow in the lower visual field (below horizon) and a focus of expansion (FOE) coinciding with current heading. (**B**) During horizontal rotation, an observer experiences uniform horizontal flow. Counter-rotating gaze at 1:1 ratio will stabilize retinal image. (**C**) Combining translation and rotation, a more complex flow is generated. Note that it lacks a FOE, and that the flow is not uniform meaning the whole flow field cannot be retinally stabilized by compensatory eye rotation. (**D**) Multiple waypoints (WP1-WP3) on the future path. Left: A waypoint follows the local visual flow. Tracking a waypoint requires counter-rotating gaze (an optokinetic pursuit eye movement). The vertical component of flow $\Delta y/\Delta t$ is proportional to the sine of vertical elevation for all points. The horizontal component $\Delta x/\Delta t$ – but for all waypoints on the circular future path it is $-1$/2 observer rotation. Right: This property follows from waypoints being fixed in the 3D scene through which the observer is moving. (**E**) Travel points on the future path and lane edge. Travel points are stable in the visual field. Fixating them implies no eye movement. The tangent point on the lane edge (TP) (cf.^10^) lies on a zero horizontal flow circle–a region of the visual flow field with no horizontal flow on a constant radius path^36^. FP$\,{\rm{FAR}}\,$ is a future path travel point in the Far Zone beyond the tangent point (cf.^12,13^). Right: Travel points, as their name sugggests, move in the 3D scene together with the observer.

The role of CEM (if any) in visual strategies for locomotion can only be decided by experiment - arguing oculomotor function from the introspection of an “unblurred” *visual field*, in absence of direct empirical evidence and rigorous theoretical assumptions about *retinal input* is clearly fraught with potential pitfalls. It is well known that a “sharp” widescreen visual field is largely an illusion, and the experience of stability of the visual field does not reflect the lability the actual retinal input^3,9^. In determining that retinal input, the movement of the eye, head and body (translation and rotation of the point of vantage) in a textured 3D environment create a complex flow field (Fig. 1A–C): the entire flow field (or retinal image) cannot be completely stabilized because the angular velocities of texture elements at different parts of the visual field are very different - thus the stabilizing eye movements’ parameters in a task will depend on the gaze strategy: in particular where in the scene gaze is directed, and whether or not scene elements are visually tracked with active eye movement (i.e. whether or not retinal slip on the fovea is compensated by gaze tracking local optic flow).

There is of course a long and strong tradition for designing optical and self-motion stimuli that reliably elicit retinal-image stabilizing CEM in the laboratory. Yet it is not well-understood whether and how these are deployed in naturalistic locomotor tasks. As opposed to the experimental stimuli and task being designed to elicit a specific eye-movement pattern of interest, the research question here is what gaze strategy is employed spontaneously?

Previous inconsistent results, especially in lab simulations, have rendered the empirical situation inconclusive. This is unfortunate, as measurement of CEM parameters provides a means to directly test quantitative predictions of different gaze-steering coordination hypotheses in the theoretical literature on visuomotor control of locomotion.

In mathematical models of visually controlled steering, the most-cited models posit online steering control based on *travel points* such as the tangent point^10,11^ or points gliding along on the future path^12–15^. Such models neither predict nor account for compensatory eye movements during locomotion, as they predict the point of fixation to travel smoothly along with the observer.

CEM are predicted by *waypoint models*^12,16,17^, as point of fixation would be approached by the observer, requiring “tracking fixations” (smooth pursuit or optokinetic response in oculomotor terms). However the most-cited waypoint models are purely theoretical proposals, the studies putting forward no direct empirical evidence, which may explain why they remain a minority view in the literature (for historical overview and theoretical discussion see^18^).

Empirically, many experimental studies of real-world locomotion have indeed documented compensatory eye movements in walking^19–21^, bicycling^22^ and driving^23,24^ – that is, fixation of locomotor targets or robust optokinetic nystagmus when locomoting on a curved path. However, other studies claim that the dominant strategy is not to track fixed locations, but instead target points that are moving with the observer using “travel fixations”^25,26^.

Worryingly, whereas a number of studies involving locomotion in the real world, on foot or driving indicate a CEM-based gaze strategy, lab studies on comparable tasks – where one would expect the most accurate and detailed measurements to reveal more oculomotor detail – may claim no tracking eye movements (e.g.^25^, or, while finding clear optokinetic nystagmus as would be produced by tracking fixations^27^ nevertheless that the *quantitative* correspondence between gaze rotation and estimated local flow was poor). The field and simulator studies thus seem to paint a divergent picture. This could indicate that the way the oculomotor strategy is organized in the real-world could in fact critically depend on vestibular stimulation and/or stereoscopic stimulation. Hence, rather concerningly, existing lab/simulator studies (with non-stereo displays and fixed base simulators) would completely lack ecological validity with regard to studying CEM mechanisms in naturalistic tasks.

A terminological note: we use the term optokinetic to indicate that gaze rotation matches local optical stimulus angular velocity–*without* committing to any hypotheses concerning the role of different signal sources, such as retinal slip, extraretinal sensory information or top-down signals. Likewise, the term “fixation” is used throughout in the sense that *gaze* is fixated to a target that can be an object or a location. Fixation therefore does not mean that the eye stays still in its socket. When targets move in relation to observer or observer moves in relation to the target or both, from an oculomotor point of view, these “tracking fixations” are actually pursuit movements. When the angular velocity of pursuit matches the egocentric motion of texture elements at the point of regard in the visual field, we say the pursuit is “optokinetic”. That is, we use this term purely descriptively, to indicate the match between stimulus and gaze rotation, and do not make assumptions about whether the movement is driven by bottom-up retinal slip reflex, extraretinal signals, top-down expectations, or any particular combination of these. (For further discussion on the conceptual differences between the literature on naturalistic gaze behavior and oculomotor control, see^28^.

## Aims of the Study

The aims of the present study were twofold. First, we wanted to gain a parametric understanding of CEM in natural, head-free locomotion when humans are engaged in an active steering task (and are not given any gaze instructions nor presented with experimenter-designated targets, other than a path to follow). The underlying theoretical motivation was the debate in the visual steering models literature between waypoint models and travel point models, such as the TP hypothesis. Second, we wanted to directly compare CEM in a similar task in the real world (with inertial cues and stereopsis) and a more typical lab-based steering task. This was in order to shed light on the diverging results in the literature, where, on the whole, field experiments tend to report evidence for CEM in active locomotion, and simulator studies against.

Could it be that the natural fixation strategy (and hence visual input to the brain) is radically different in real-world and in simulated locomotion, when the latter are lacking stereopsis and inertial cues? This would clearly place a severe limitation for the possibility of studying this natural visuomotor gaze-locomotion strategy in lab settings. On the other hand, if reliable and systematic OKN were found, this would suggest that i. the spontaneous gaze strategy is to track waypoints on the future path, ii. that this strategy is not critically dependent on stereopsis or inertial cues, and iii. the lack of results in previous work might be due to methodological issues that need to be taken into account in any future careful studies of gaze-locomotor coordination (such as display technology, stimulus materials or analysis techniques).

**Figure 2:**
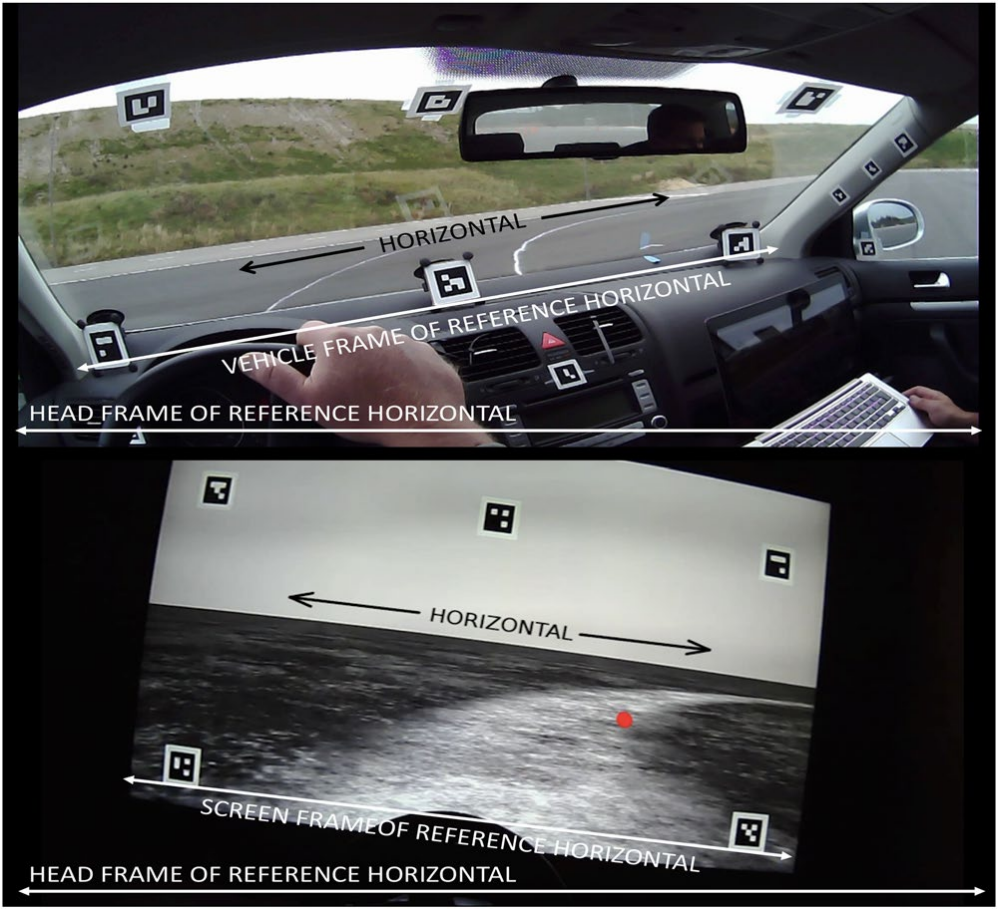
A view of the head-mounted eye tracker scene camera in the test track experiment (top) and simulator experiment (bottom), showing the participant’s view of the constant radius (circular) path. Red dot indicates point of regard. The eye tracker calibrates eye images to the head frame-of-reference. As the participant rolls her head, the world horizontal axis is tilted. To estimate horizontal visual flow – i.e. horizon of the plane of translation – the screen horizontal axis in the simulator, and the car’s horizontal axis on the test track were defined based on the optical visual markers seen on the windcreen, dashboard and screen image. (Text and arrows not visible during experiment).

Two experiments were run: one in a fixed base simulator and one in a test track environment. The same “friction circle” task was performed in both experiments: participants (N = 4 in the test track experiment, N = 15 in the simulator experiment) drove on a circular path with a constant radius, with increasing speed. In a constant radius curve yaw rate is directly proportional speed. Thus, we were manipulating observer rotation rate as the independent variable, and observing gaze counter-rotation as the dependent variable. Observer rate of rotation was obtained from vehicle telemetry and the simulation software. Gaze direction was measured using a wearable head-mounted eye tracker, and projected to the locomotor frame of reference – i.e. vehicle (test track) or screen (simulator) coordinates – using optical markers in the forward looking camera image (Fig. 2; for detailed methods see the Methods section).

Based on theoretical literature, we were able to make very specific predictions:

1. If the natural visual strategy of the participants is to track waypoints on the future path, then *gaze horizontal velocity and vehicle yaw rate should correlate linearly; specifically, gaze horizontal velocity would be* $-1$/2 of the car’s yaw rate^16,17^. If, on the other hand, the spontaneous visual strategy is to track travel points, such as tangent points^10^ or points on the future path at a fixed distance^13^, then *gaze horizontal velocity should be constant, at zero, and not depend on the car’s yaw rate*. The rationale for this prediction is the following: Consider an observer travelling on a constant radius trajectory on a textured flat terrain. Here, the angular horizontal visual flow speed at the future path (or equivalently the horizontal angular velocity of a waypoint on the future path) can be geometrically shown to be negative of half of the observer’s yaw rate^16,17^ (Fig. 1D; for an intuitive version of the argument see Supplementary Materials). Hence, when looking at a static point of fixation on the path, gaze wil counter-rotate at $1/2$ observer rate of rotation. This is not the case when the observer fixates a travel point, such as the Tangent Point or a point at a fixed distance ahead on the Future Path (as posited in the models of e.g.^10,11,13,14^ (Fig. 1E). These visual targets are not fixed to the world, but move in the plane of travel with the observer. Hence, when gaze is fixed on them no gaze rotation is seen in the observer frame of reference:the gaze vector is “rigidly fixed” to the observer and carried in the world with observer locomotion.
2. If the waypoint tracking is indeed the predominant strategy, and the compensatory pursuit movements are driven by the moving optical pattern (local flow), then *simulator and real life experiments should give quantitatively or at least qualitatively similar results*. But if stereoscopy or vestibular sensations are critical to organizing the oculomotor strategy for these CEMs, then the correlation will only be observable in real driving - or at least it will be much attenuated in the simulator.

## Results

**Figure 3:**
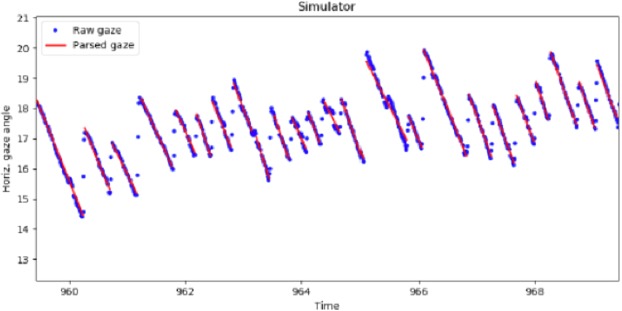
Pursuit identification from gaze position data. Time series graph from an example clockwise run–i.e. turning right–in the simulator experiment. Horizontal (in screen reference frame) gaze position is plotted on the y axis against time on the x axis. Positive y values indicate direction to the right. Red lines are linear segments fitted with the NSLR algorithm to identify the tracking fixations – i.e. parse the raw data into pursuit segments. The linear segment slope at each time point was used as the horizontal gaze rotation estimate.

It is immediately clear in both experiments that the horizontal gaze position pattern (Fig. 3) shows optokinetic nystagmus (OKN) with pursuit-like slow phases (SP, gaze rotating right to left in a clockwise right-hand turn) and saccadic quick phases (QP, gaze jumping left to right). Individual fixations (actually OKN SPs) were identified using a denoising and event identification algorithm designed to detect smooth pursuit movements from both field and lab data^29^. We estimated the horizontal velocity component of gaze as the slope of the linear segments fitted to the gaze position time series data points.

**Figure 4:**
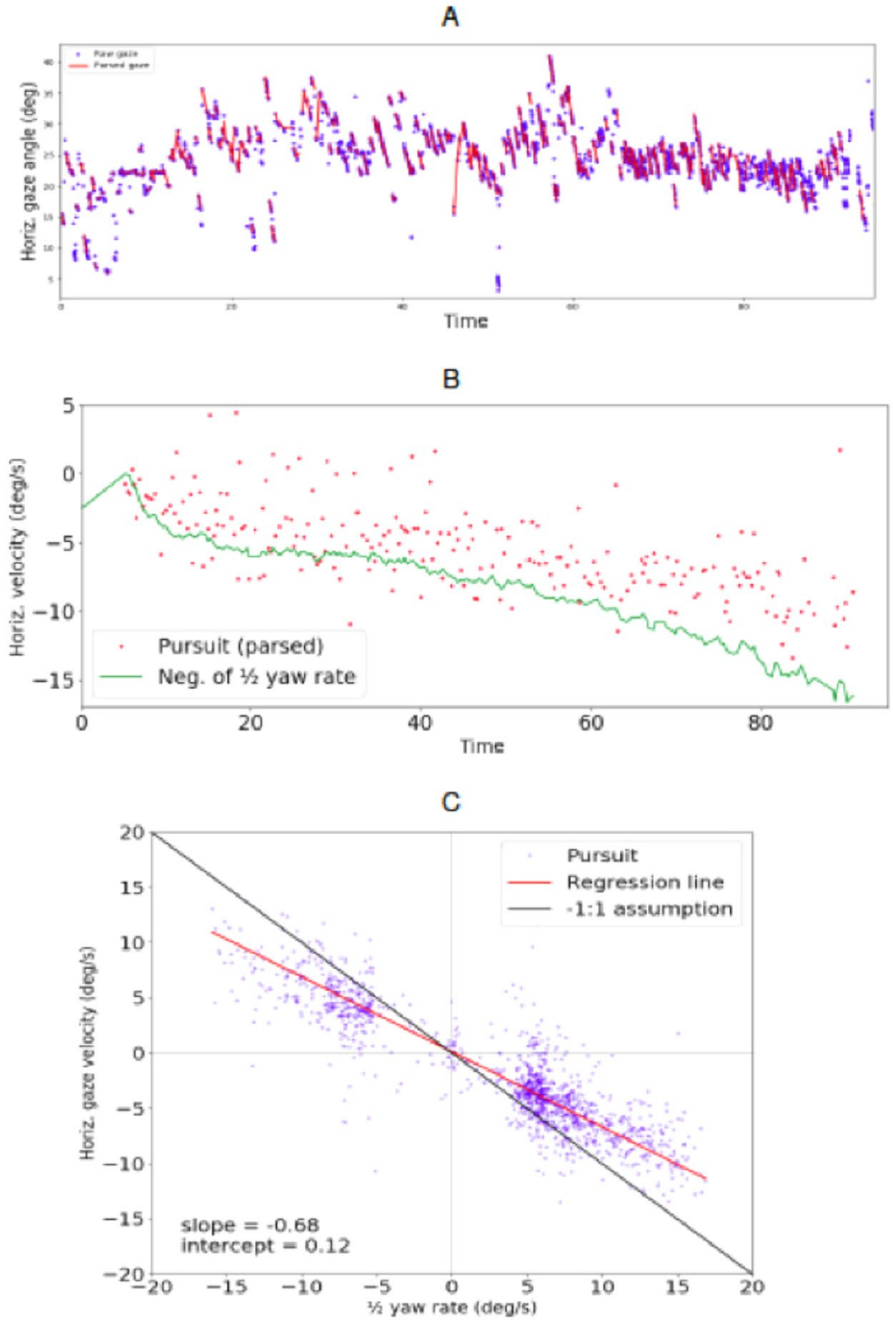
Typical individual participant data from the test track experiment. Top. Horizontal gaze angle time series from one. Blue dots are raw gaze positions, red lines the linear segments fitted by the fixation identification algorithm. Middle. Pursuit slopes i.e. gaze velocities (red dots) and negative of half yaw rate (green line) from the same trial. Bottom. All data from the individual participant. Blue dots are individual pursuits plotted against average yaw rate at the time of the pursuit. Red line is orthogonal regression fitted to the data. Black diagonal indicates $-1$:1 prediction.

**Figure 5:**
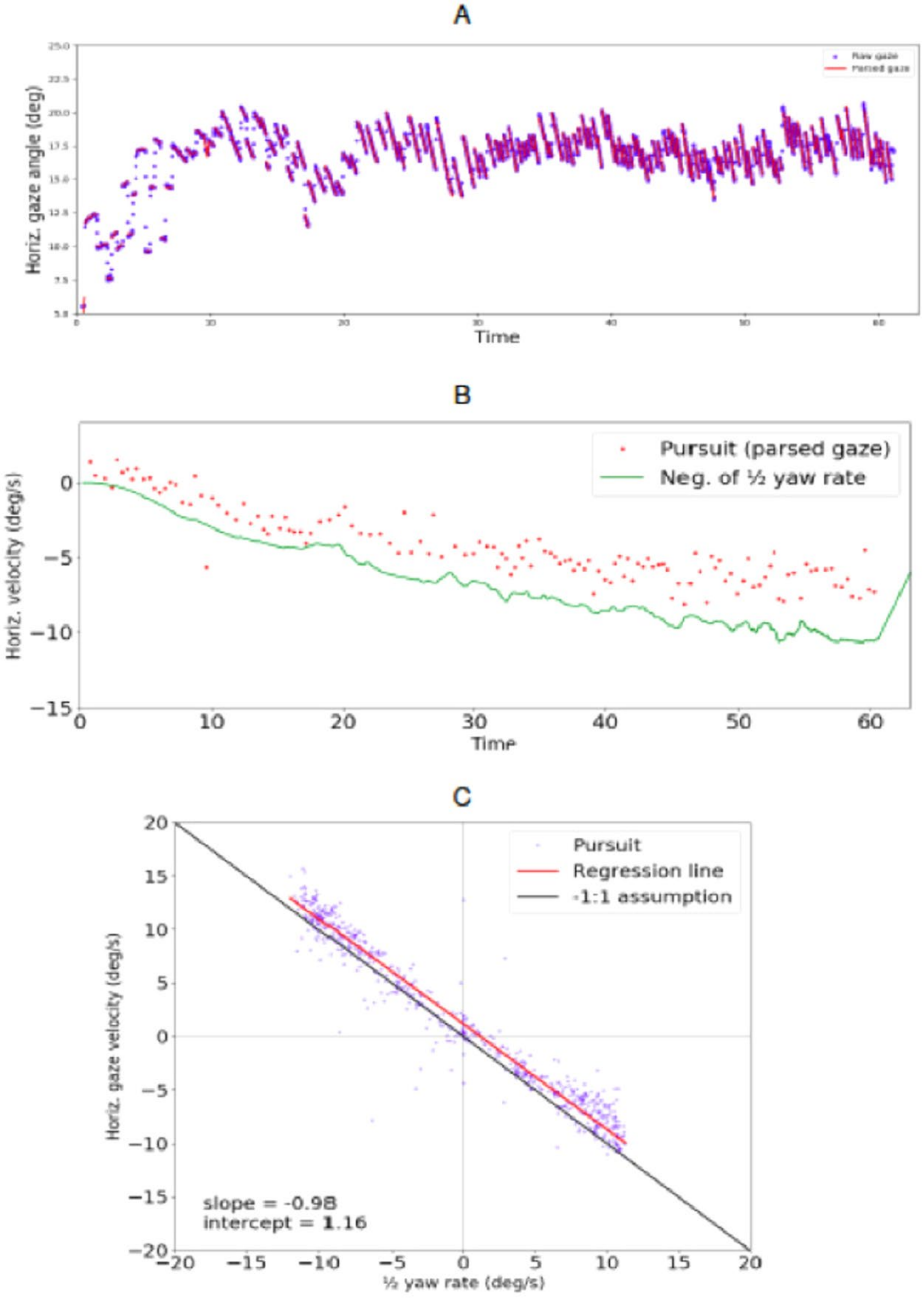
Typical individual participant data from the simulator experiment. Top. Horizontal gaze angle time series from one. Blue dots are raw gaze positions, red lines the linear segments fitted by the fixation identification algorithm. Constant acceleration of the car causes the rate of rotation to increase, and the effect of this can be seen as steeper slopes for the pursuits later in the series. Middle. Pursuit slopes i.e. gaze velocities (red dots) and negative of half yaw rate (green line) from the same trial. Bottom. All data from the individual participant. Blue dots are individual pursuits plotted against average yaw rate at the time of the pursuit. Red line is orthogonal regression to the data. Black diagonal indicates $-1$:1 prediction.

Typical individual participant results from the test track and simulator experiment are given in Figs. 4 and 5, respectively. The top panel shows an individual trial horizontal gaze position time series, displaying a very clear and periodic optokinetic nystagmus in both cases. The slow phase gaze velocity (and frequency of the nystagmus) appears to increase with time as the vehicle accelerates and rotation rate increases. Comparing observer and gaze rotation allowed us to assess the quantitative relationship between locomotion and the compensatory gaze counter-rotation. The middle panels in Figs. 4 and 5 show yaw rate (i.e. observer rotation) gaze horizontal velocity (i.e. gaze rotation) example time series. Both increase in step with one another over time, as the vehicle is accelerated on a constant radius path. In the bottom panel, a scatterplot shows yaw rate and gaze velocity data from all the runs of the subject (a data point is individual tracking fixation, i.e. identified pursuit). Corresponding plots for all subjects are given in the Supplementary Results.

As a (near) linear correlation is predicted by the waypoint hypothesis, the Pearson correlation coefficient was chosen to estimate gaze-locomotion correlation (Spearman’s and Pearson’s correlation coefficients were compared within each subject but differences were all under 0.025 in both experiments, which in itself indicates linear correlation).

Each algorithmically identified pursuit segment was a data point to be correlated with interpolated yaw rate at each timestamp. In the test track experiment, correlations were strong (subject median $-0.74$, mean $-0.77$, 95% confidence interval [$-0.85$, $-0.70$]). In the simulator experiment, data showed a very strong correlation for all participants (subject median $-0.93$, mean of $-0.92$, 95% confidence interval [$-0.96$, $-0.87$]).

The Pearson correlation coefficient indicates linear correlation but does not give the numerical ratio of the variables. As the linear correlations are high, it is reasonable to conduct a linear regression analysis. We used orthogonal regression which differs slightly from simple linear regression. Orthogonal regression finds the best-fitting linear regression line for the data by minimizing the distance from the data points to the regression line just like traditional linear regression, but it calculates the distance from data point to regression line in both x- and y-axes (where simple linear regression uses just the y-axis). This avoids regression dilution, i.e. systematic underestimation of the slope’s magnitude due to measurement noise in the x-axis value (ie. the yaw rate).

**Figure 6:**
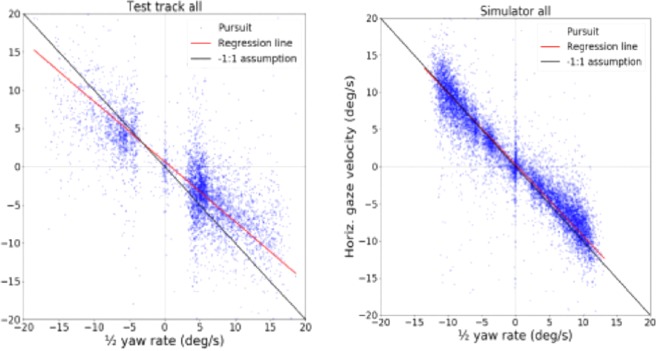
Fixation-level horizontal gaze velocity (i.e. NSLR identified pursuits) plotted against $1/2$ yaw rate. All participants’ data. Left. Test track experiment (n = 4) Right. Simulator experiment (n = 15). Orthogonal regression line (red) is close to the $-1$:1 prediction (black diagonal). This is especially true for the simulator data; the test track data has less data points in the lower speeds region and overall shows more variance than simulator data.

Figure 6 shows fixation-level gaze data (i.e. each data point is a single pursuit movement) against yaw rate, pooled across all participants. The test track data indicate a strong negative linear correlation ($-0.75$) and the simulator experiment data a very strong negative linear correlation ($-0.91$). Test track data and simulator data’s and regression slopes for aggregated data were $-0.79$ and $-0.96$, respectively. Intercepts were 0.66 deg/s (test track) and 0.32 deg/s (simulator).

The regression slope tells us the ratio the two correlating variables. The waypoint hypothesis predicts a $-1$:1 ratio (i.e. $-1.0$ slope and 0.0 intercept) between half yaw rate and horizontal gaze velocity. Travel point hypotheses, such as the tangent point hypothesis, predict zero slope and zero intercept. The regression line parameters for individual participants are given in Table 1. Every participant in the simulator experiment clearly had a slope very near the predicted value (subject regression slope 95% CI [$-1.01$, $-0.91$]; median $-0.96$, mean $-0.96$), and the same was true (albeit somewhat less clearly) in the test track experiment (subject regression slope 95% CI [$-1.13$, $-0.53$]; median $-0.72$, mean $-0.83$). Median intercept in the simulator experiment was 0.19 deg/s (subject mean 0.31 deg/s and 95% CI [0.02, 0.60]). Median intercept in the test track experiment was 0.71 deg/s (subject mean 0.079 deg/s and 95% CI [$-0.10$, 1.68]).

**Table 1:** Horizontal gaze rotation rate vs. $-1$/2 vehicle rotation rate orthogonal regression parameters for individual participants.

Test track experiment	Participant	Regression slope	Regression intercept
	1	$-0.68$	0.12
	2	$-0.6$	$-0.7$
	3	$-1.28$	1.3
	4	$-0.76$	1.8
	All	$-0.79$	0.66
**Simulator experiment**	**Participant**	**Regression slope**	**Regression intercept**
	1	$-0.98$	1.18
	2	$-1.02$	$-0.31$
	3	$-1.15$	$-0.3$
	4	$-1.03$	0.17
	5	$-0.79$	0.52
	6	$-0.85$	0.94
	7	$-1.11$	0.19
	8	$-0.94$	$-0.48$
	9	$-0.88$	0.8
	10	$-0.89$	0.51
	11	$-0.96$	0.2
	12	$-0.97$	0.32
	13	$-1.03$	0.59
	14	$-0.9$	$-0.23$
	15	$-0.88$	1.46
	All	$-0.96$	0.32

The field experiment participants were expert drivers, and took part in the field experiment before the simulator experiment. To evaluate whether the participants who had taken part in the field experiments behaved similarly to the others, simulator experiment data of the participants who participated in both experiments (n = 4), were compared to those who drove only in the simulator experiment (n = 12). Gaze velocity-yaw rate Pearson correlation coefficient for participants who participated in both experiments had a median value of $-0.92$ (mean $-0.94$, 95% CI [$-0.97$, $-0.90$]), and for participants who only took part in the simulator experiment a median value of $-0.94$ (mean $-0.91$, 95% CI [$-0.97$, $-0.85$]). Orthogonal regression slope median for participants in both experiments was $-0.90$ (mean $-0.93$, 95% CI [$-1.03$, $-0.84$]). For participants who drove in the simulator experiment only the median regression slope was $-0.97$ (mean $-0.97$, 95% CI [$-1.02$, $-0.91$]). As 95% confidence intervals overlap with both slope and correlation, these results do not suggest any statistical difference between these participant groups in correlation or regression slope.

## Discussion

Two experiments on active gaze in hihg-speed steering were run to determine to quantitatively analyze spontaneous (non-instructed) optokinetic eye movement behavior during a head-free conditions. Observer rotation (locomotor speed) was manipulated as the independent variable, and the effect on gaze counter-rotation was estimated by parsing the gaze signal into pursuits segments using an eye movement event detection algorithm designed to work with data collected in challenging conditions^29^. This allowed us to state the quantitative form of the relation of active gaze and self-motion under naturalistic conditions of curvilinear locomotion.

One experiment was run in an instrumented vehicle on a test track, and the other in a fixed base driving simulator. Using both a real car and a fixed-base simulator set-up we were able to directly compare the spontaneous gaze strategy in conditions where stereoscopic cues and non-visual motion-stimuli are present (real car) and absent (fixed base simulator), as this could potentially explain some of the discrepancies between previous reports.

The most important methodological aspect of the field-lab cross-validation paradigm was thus to see whether the correspondence between observer rotation and compensatory gaze movement would hold in a fixed base simulation where there is only visual stimulation (and of course motor efference copy)–but no stereoscopic depth information, inertial vestibular cues, or “seat of the pants” somatosensory acceleration or haptic steering feedback. Specifically, should we observe OKN in the field experiment only, we would then have had to conclude that either stereo cues or extraretinal signals or both are critical for accurate visual tracking in locomotion, which would imply grave consequences for the ecological validity of typical visual steering and driving simulator experiments on visuomotor coordination). Contrary to some previous claims, we did observe highly accurate tracking in the lab. From this we can conclude that the nystagmus is indeed “optokinetic”, i.e. can be elicited in situations where the only self-motion cues are visual, and therefore profitably studied with typical laboratory set-ups.

Theoretically, our experimental task allowed us to investigate whether the gaze strategy in constant radius curve driving is fixation of a travel point, as suggested by some mathematical steeering models and experimental studies, or fixating waypoints with the help of compensatory eye movements. Waypoint models posit that gaze is fixed on locations on the future path that the observer desires to pass over, and from geometric considerations it can be shown that during combined translation and rotation on a constant radius path this predicts negative linear correlation; more specifically a $-1$:1 ratio between horizontal gaze velocity and half of observer locomotor yaw rate when gaze counter-rotation observations are correlated with observer rotation across a range of speeds. The tangent point hypothesis on the other hand^10,11^, and other travel point models such as models based on pursuing a future travel point at a constant distance ahead^13^ predict zero correlation between gaze rotation and yaw rate in the these conditions.

The results from both experiments unequivocally support the waypoint hypothesis over the travel point hypothesis. In both experiments, the horizontal gaze position exhibited a clear “sawtooth” OKN pattern for every participant when observed as a time series. Slow phases of optokinetic nystagmus (pursuit movements) were identified, the horizontal gaze velocity component estimated, and correlated with observer horizontal rotation across a range of velocities. As predicted by the WP hypothesis, the horizontal component of gaze velocity and (half of) observer yaw rate showed a strong linear negative correlation ($-0.75$ in the test track experiment, $-0.91$ in the simulator experiment). Regression analyses per subject showed slopes consistent with the predicted $-1$:1 ratio between the relevant variables (test track 95% CI [$-1.13$, $-0.53$]), simulator 95% CI [$-1.01$, $-0.91$]). The pattern can be clearly seen even on individual level, and is the same for every participant (see Supplementary Results).

### Implications for oculomotor research

Smooth pursuit, vestibulo-ocular and optokinetic responses, and the accompanying head movements offer a suite of basic visuomotor coordination patterns that can be used between saccades to stabilize the retinal image during locomotion. Indeed, it is not uncommon for oculomotor researchers to unproblematically assume this is so. That is, SEM such as optokinetic response, smooth pursuit and vestibulo-ocular response are generally considered to contribute the retinal image stability, and referred to as “compensatory” CEM. Understanding CEM in the wild is theoretically significant because the visual strategies directly influence the actual pattern of retinal input received by the brain (and image-stabilizing movement also produces an efference copy that can be used by central mechanisms for visual stability). Laboratory studies often begin with an introductory vignette outlining how the mechanisms under study could contribute to natural task performance – but there is a dearth of empirical data (and citations) actually assessing the comparability of the naturalistic tasks (where participants are typically engaged in some primary task, and no eye movement instructions are given) and analog lab tasks.

Our data unequivocally show not only robust OKN but remarkably clear match between observer rotation and gaze counter-rotation in a simple steering task in a typical fixed-base setup. This means that compensatory eye movement patterns in active locomotion is not critically dependent on stereoscopic or inertial cues, and can be fruitfully studied in fixed base simulator settings – provided care is taken in design of the optical stimuli and analysis of eye-movement data.

### Relation to previous research

How do our results fit with the somewhat inconsistent picture painted by previous studies? In the study of spontaneous gaze strategies (in tasks such as driving), the empirical situation on the presence and characteristics of CEM has in fact been much more muddy than one might expect. What is more, field and laboratory results seem to be diverging rather than converging, Field studies of walking^19–21,30,31^ bicycling^22^ and driving^23,24,32^ tending to report CEM (thus qualitatively our result of robust OKN in the test track experiment complements these existing observations – though for a diverging view see^26,33^). But laboratory studies – where one would expect the most accurate and reliable behavioural and psychophysiological measurements to come from – tend to report either no OKN^25^ or OKN poorly matching visual flow in simulated locomotion^27^. (Qualitatively and quantitatively in contrast to present results). This is concerning, because it could suggest that the more restricted stimuli and task environments of simulated locomotion might be insufficient to elicit the natural CEM-type gaze strategy in lab studies, bringing to dispute the external validity of studies of simulated locomotion in general, and simulated driving in particular. How are the inconsistencies to be resolved, and what kind of a way forward is indicated by the present results?

Authié & Mestre^27^ and Lappi & Lehtonen^23^ independently discovered OKN in simulated and real curve driving, respectively. Itkonen *et al*.^24^ directly compared gaze rotation to the vehicle yaw rate (rate of change of heading) and found good agreement with the value predicted by waypoint tracking. That is, gaze counter-rotated at the $-1$/2 ratio to vehicle. (These experiments were performed on a motorway on-ramp at a fairly constant speed and thus constant yaw rate). In contrast, Authie & Mestre^27^, in their simulator study, found poor quantitative correspondence between gaze velocity and estimated local visual flow (i.e. the projection on the screen of a fixed scene location where gaze was estimated to land). What is more, other lab experiments report that no tracking eye movements are present in the data^25^ and some earlier field experiments claimed this as well^26^.

We can immediately point out, however, that on methodological grounds the null results of Wilson *et al*.^25^ and Kandil *et al*.^26^ are actually quite weak. The authors only state they do not “observe” pursuit movements, but individual pursuits are not all that salient when looking at a scene camera overlay video. The papers did not actually show any raw time series data – and did not calculate informative quantitative measures or statistics. Given how clear the OKN^27^ time series figure highlights how important it is to represent data in the right coordinate system.

What, then, could explain the difference between our Experiment 2 and Authie & Mestre’s laboratory experiment, where the predicted correspondence between gaze velocity and estimated point of regard angular velocity was according to the authors, not present. Their specific finding was that the correspondence between gaze movement and visual flow direction areas of interest placed around the point of regard (letting AOI size range between 0 deg and 7 deg, and the whole screen) was poor. However, when one considers the pattern of flow on a curvilinear path (Fig. 1) it is clear that a very large AOI will contain elements with flow in many directions and varying magnitudes. Ideally, only rather local flow around waypoints is approximately at $-1$/2 yaw rate. For these smaller AOI sizes, it is important to look at the method they used to evaluate the visual flow. They did this by first projecting gaze to the simulated scene and then estimating the visual motion of the point of fixation by *projecting it back to screen coordinates*. This is not a very robust method, as even slight error in estimated gaze position (e.g. from drifting calibration) can change the point of fixation to one that has very different motion from the motion of the actual target. The conceptually more indirect way used in our study (correlating horizontal gaze velocity to observer rotation) is much more robust, and not affected by calibration bias. This highlights the importance of analyzing the data using measures that are at the same time informative and robust.

Other potential sources of differences are textures and equipment. We took pains to create textures and render them in a manner that do not produce pronounced aliasing or blurring. 3D rendering of textures at steep angles, which occur when a planar road environment is projected for the driver, is a somewhat challenging task. To minimize aliasing and blurring, it is crucial to use high resolution textures with high quality anisotropic sampling.

Finally, but not the least insignificantly, one must consider the task. The Authie and Mestre^27^ instruction was to drive as fast as possible without driving off, implying hard acceleration and braking, which might change the gaze behavior. Also the Wilson *et al*.^25^ study used a racing game. It would be interesting to investigate further whether these differences in steering strategy could have make the gaze strategy qualitatively different.

## Conclusions

The contribution of active gaze strategies to visual stability and perception of an “unblurred” visual field in naturalistic locomotion remain a surprisingly little researched topic. Laboratory experiments probe eye control in sedentary, usually even head-restrained conditions, and with highly reduced stimuli specifically designed to elicit a particular type of movement of the eyes. It is commonly thought that in richer naturalistic tasks visual stability in part rests on the same or similar SEM mechanisms working in synergy with each other (and head- and body-stabilizing movements). But it is still not known precisely how or whether the models of oculomotor control derived from restricted tasks apply to natural gaze behavior.

The oculomotor basis of CEM has been extensively studied in laboratory research, but the literature regarding their presence and properties during locomotor tasks is not entirely consistent. Many field studies have documented optokinetic pursuit movements in walking, bicycling and driving^19–24^. However, some^26^ have denied any was present, and lab research on simulated driving has often reported that the quantitative correspondence between gaze rotation and optic flow is poor^27^, or even that no tracking eye movements are present^25^. This could indicate that the way this “optokinetic” oculomotor strategy is organized in real-world driving could be critically dependent on vestibular stimulation and/or stereoscopic stimulation, which would imply limitations for the possibility of studying this natural visuomotor gaze-locomotion strategy in the current generation of lab-based steering tasks or simulators.

Our results from two experiments - one in a fixed base simulator and one in a real car on a test track environment – where participants drove on a circular path with a constant radius, with increasing speed show an unambigous correlation between observer rotation and counter-rotation of the gaze. (Eye movement parameters were accurately estimated with an event detection algorithm specifically designed for use with the data collected in challenging conditions, rather than recoursing to visual inspection). This shows vestibular or stereo input is not essential for spontaneously adopting this gaze strategy – suggesting that failure to observe OKN in some previous simulator studies are likely due to methodological shortcomings, rather than different gaze strategies in simulated and real locomotion.

Second, our results speak directly to the debate in the modelling literature on whether the natural gaze targets in locomotion are travel points or waypoints. When tracking waypoints on the future path, guiding “fixations” are actually slow pursuit eye movements (“fixation” meaning that gaze is fixed on a target, rather than fixed in the head frame of reference – these definitions will diverge whenever the observer is in motion). The present results thus strongly support the contention that the primary gaze strategy when cornering in a curve is indeed to pursue local flow/waypoints on the future path, at least across a range of rotation up to 20–30 deg/s. This is inconsistent with the TP model and other travel point hypotheses, but consistent with the waypoint identification hypothesis that has been put forward as a general locomotor strategy.

Visual control of locomotion is a complex task with multiple sophisticated visual strategies that are only beginning to be understood. Yet the task is fundamental and general – and best of all sufficiently stereotypical subtasks can be isolated for rigorous scientific analysis even “in the wild”. While it is important to be able to break complex performance down and simplify it into subtasks that can be isolated and analyzed, in order to gain real understanding of actual performance it is also necessary to be able to put the pieces back together, and show that the isolated (e.g. oculomotor) patterns of behavior indeed show up under more natural task conditions. Tasks where this can be done offer opportunities to bridge simplified laboratory, high-fidelity simulation and real-world tasks in a way that very few behavioral, let alone psychophysiological, experimental paradigms can offer.

## Methods

The test track and simulator experiments were designed to be as similar as possible, within practical limitations. The main points of difference were: (i) for maximal experimental control in the simulator the driver’s speed was controlled by the computer. We did not have the equipment to place speed under experimenter control at the test track, and thus the drivers themselves controlled the speed. (ii) the curve radius was bigger in the simulator (where it is safe to use higher speeds) and (iii) there were some visual differences to the layout of the simulated path (explained in detail below).

### Ethics

The study was approved by the ethical review board of the University of Helsinki, and followed the Helsinki declaration and guidelines of the Finnish committee for research ethics (www.tenk.fi). Upon arrival the participants were debriefed as to the purpose of the study, and they signed an informed consent form for the publication, and the use of the collected data for scientific purposes.

### Participants

The participants’ background information statistics are shown in Tables 2 and 3. All participants had normal or corrected vision, reported no eye-movement related neurological conditions or medication, and held a valid driver’s license. All participants were naive to the specific hypotheses of the study, and were not told about the theoretical difference between travel points and waypoints.

**Table 2:** Test track experiment participants.

N	Age	Self-reported driving experience	
		Past 12 months	Total
4 (4 M)	Range 24–39 y, median 28 y	Range between 15000–30000 km, Median between 15000–20000 km	Range between 100000–500000 km, Median between 100000–300000 km

**Table 3:** Simulator experiment participants.

N	Age	Self-reported driving experience	
		Past 12 months	Total
15 (9 W 6 M)	Range 24–46 y median 28 y	Range between 1000–30000 km, Median between 5000–10 000 km	Range between 1000–500 000 km, Median between 30 000–100 000 km

Participants for the test track experiment were recruited through personal contacts by AT. The recruiting process took into account that the experiment required good driving skills so that the subject could handle the car at speeds approaching the limit of lateral grip, and also control the speed according to instruction. (Originally, eight participants drove the driving task. However, information about neurological conditions potentially affecting the eyes - such as strabismus - came to light for two participants after the experiment. One participant had poor calibration and also failed to follow task instruction. Finally, car telemetry data from one participant was lost due to a computer error, leaving a dataset of four participants).

Participants for the simulator experiment were recruited through university email lists, except for three, who also took part in the track driving experiment and were recruited by AT. These three participants were useful to see if eye behaviour was consistent between the experiments, and whether these semi-expert participants’ gaze strategies (in both experiments) would be comparable with population-normal subjects without specific driving skills.

### Eye tracking methodology

In both experiments eye tracking was done with the same system, and as much as possible using the same post-processing workflow, as this ensures maximum comparability between the lab and field. We used the Pupil Labs Binocular 120 hz eye tracker. (Pupil Labs UG haftungsbeschränkt, Berlin, Germany). Eye image data was recorded in 30 Hz at 640 $\times$ 480 resolution. Headset stability was improved with a custom headband (see Supplementary Methods).

**Figure 7:**
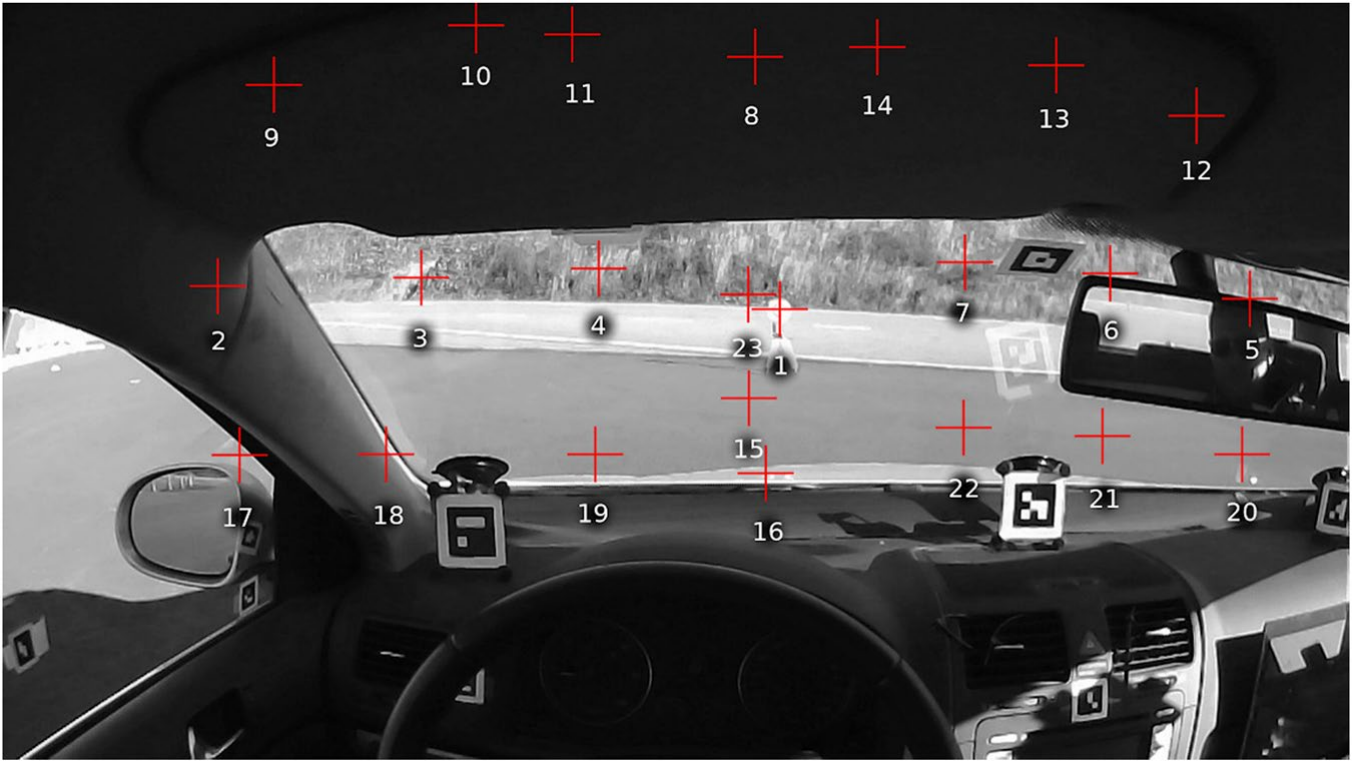
An example of typical locations of the calibration points in the driver visual field (red crosses). Instead of multiple targets, we used a single target on a tripod about 7 m in front of the car (calibration point 1), and the participant was instructed to turn their head and hold fixation in a pre-determined sequence, which brought the calibration target into different parts of the head-referenced visual field.

When calibrating the eye tracker in the track experiment, the participant was instructed to move their head in different poses while maintaining fixation on the target. This moved the fixed gaze target to different parts of the visual field of the scene camera (Fig. 7). For each calibration 22–24 calibration points were recorded. If calibration was deemed to have drifted (likely due to headset movement) a new calibration was performed. The rule of thumb was that judged positional accuracy error should be under 2 degrees in the central region. Optical markers for estimating head pose in the vehicle frame of reference were placed on the dashboard, windscreen, A-pillars and side windows (visible Fig. 7, see also Supplementary Methods).

In the simulator experiment, two calibrations were made during the collection of the present data (the participants completed a number of other driving tasks not reported here, and there were further calibration checks preceding and following them). Participants fixated on sequentially appearing stationary markers with their eyes. Calibration accuracy was measured using difference between measured gaze and target point in the frames of the next calibration sequence. Median accuracy during calibrations was 1.0 degrees (variance 0.47), median horizontal accuracy 0.59 degrees (variance 0.25) and median vertical accuracy 0.70 degrees (variance 0.40).

### Instrumented vehicle

The instrumented vehicle was model year 2007 Volkswagen Golf V Variant 1.9TDI with manual transmission. Vehicle telemetry was recorded from CAN-bus with 100 Hz sample frequency.

### Test track site

The test track experiment took place at Vantaan Ajoharjoittelurata driver training track in Vantaa, Finland (N 60.334139, E 24.927111). The weather on the test days was dry and warm.

The friction circle outer diameter was 25 m and inner 22 m. The diameter was chosen to be as big as possible considering the surroundings and safety. It was marked on the asphalt with chalk spray, which was chosen to draw track edges as it has several advantages over other options such as cones: it is flat, has no distinctive 3D features to fixate eyes on, it is safe to run over, removable and sufficiently easy to use.

### Test track procedure

Upon arrival the participants were briefed about the experiment and signed an informed consent form and answered a background information questionnaire.

Eye tracker calibrations were performed before and after the test. In the car during the test were the participant, the experimenter operating the eye tracker and giving the instructions for calibration and task (PR), in the passenger seat, and in the back-seat a member of the research team handling vehicle telemetry data collection (AT). Questions were permitted during the test, but precise information about the studied phenomena were given only after the experiment.

The task was to “drive along the track” with a steadily accelerating speed up to the limit of friction. No instructions on gaze strategy were given. The participants performed a total of six runs, 4 runs clockwise and 2 runs counter clockwise (We chose to do more right hand turns as the A-pillars of the car blocked part of the view when turning left - in a left-hand drive car the road is better visible when turning to the right, i.e. clockwise, whereas turning left the left A-pillar obstructs the view somewhat, and sometimes the driver would be looking through the side window). Each run was driven until the maximum achievable speed was sustained for at least one full revolution of the friction circle. All participants drove to the car’s limit and none expressed feelings of anxiety (indeed they were quite enthusiastic). Further task corrections and clarifications were given between runs if needed, especially to maintain as smooth and steady acceleration as possible. Each run took between 84 and 101 seconds with median of 92.0 seconds.

After the experiment was over, the participants were debriefed, and two sports and cultural activity vouchers worth 10 € were given as a compensation of the participant’s time. Upon debriefing the test track experiment, the participants were told we were interested in “small involuntatry reflex movements” elicited in cornering, but the precise nature of these movements was not discussed to maintain them somewhat naive for participation in the simulator experiment. Total time of the experiment was approximately 50 minutes per participant.

### Simulator set-up

The physical set-up of the simulator is shown in Supplementary Methods. The steering wheel (Logitech G25, Logitech, Fremont, CA) was fastened to a table at 62 cm height, and the screen was placed directly in front of the participant. The pedals were not used in this experiment as speed was controlled by the simulation.

### Stimuli

The virtual track 50 m radius full circle, with path width 2.35 m and 0.62 m fading in both sides (path edges were faded out rather than presented as edge lines). Path width was chosen to be quite narrow but to have some room for comfortable driving.

The simulation was run at $1920\ \times \ 1080$ pixel resolution in 60 Hz frame rate. Virtual camera eye height was set to 1.6 m. Simulated engine noise was presented through the screen’s speakers. Path and ground texture were generated using “Brownian noise” (beta = 2.0) as a texture element^34^. This texture was chosen after extensive piloting because it can present patterned information in different resolution frequencies and does not produce aliasing patterns that would be seen as stationary targets on the screen. It also presents no distinctive or semantically meaningful targets.

### Simulator experiment procedure

The simulator experiment was conducted at the TRUlab at the University of Helsinki Helsinki. Upon arrival the participants were briefed with relevant information about the experiment and they signed an informed consent form and filled a background information questionnaire. Questions were permitted during the test, but precise information about the studied phenomena were given only after the experiment.

There were in total of four different tests in the experiment but only data from the third test (replicating the test track friction circle task) is reported here. The other data will be reported in a separate study. Total drive time for completing all tests was approximately 12 minutes. In total the experiment took 30 to 40 minutes. After the experiment participants were given a sports and cultural activity voucher worth 5 € as compensation.

There were no instructions other than to “drive along the track” and stay on the path. Specifically, participants were not instructed to follow the middle of the path as explicit instructions could change gaze patterns nor were they instructed on gaze strategy (cf.^24,35^).

The vehicle accelerated from 0 to 73 km/h at a constant acceleration of 0.34 m/s/s, producing rotation speeds from 0 to 23 degrees/s. One trial lasted 60 seconds and was repeated twice in each direction – i.e. four times in total. (Short 1 minute trials were used to avoid fatigue and left and right turns were altered to minimize the chance of simulator sickness and dizziness).

## Supplementary information

Supplementary Information.
