# Supplementary material for: Humans use Optokinetic Eye Movements to Track Waypoints for Steering
Source: Sci Rep. 2020 Mar 6;10:4175. doi: 10.1038/s41598-020-60531-3 (PMC7060325; doi:10.1038/s41598-020-60531-3)
Supplement: Supplementary file 1 — Supplementary Information. [file 41598_2020_60531_MOESM1_ESM.pdf]

SUPPLEMENTARY INFORMATION

for

Humans use Optokinetic Eye Movements to Track Waypoints for Steering

Otto Lappi[1,2 †S], *Jami Pekkanen[1,2 †L], Paavo Rinkkala[1,2.3 †], Samuel Tuhkanen[1,2], Ari Tuononen[4], Juho-Pekka Virtanen[5]

[1] Cognitive Science, Department of Digital Humanities & Helsinki Centre for Digital Humanities (Heldig), University of Helsinki, FINLAND
[2] TRUlab, University of Helsinki, FINLAND
[3] Spatial Planning and Transportation Engineering, Dept. of Built Environment, Aalto University, FINLAND
[4] Department of Mechanical Engineering, Aalto University, FINLAND
[5] Aalto University School of Engineering, FINLAND

Supplementary Discussion

Geometrical basis for the gaze velocity prediction from the waypoint hypothesis

An intuitive way to see the -½ ratio between observer rotation and gaze counter rotation when tracking a waypoint is as follows. Let there be a path that, when followed, rotates the observer by n degrees, say 90 degrees **(Supplementary Figure S1)**. The angle of gaze and current heading will be n/2 degrees (e.g. 45 degrees, see illustration). Keeping the point of fixation (i.e. end point of the gaze vector) on the waypoint at the end of the path, gaze must counter-rotate so that at the moment the waypoint is reached the gaze is again aligned with the direction of heading at the end of the path (arriving at the waypoint). By this time the gaze will have counter-rotated -n/2 degrees.

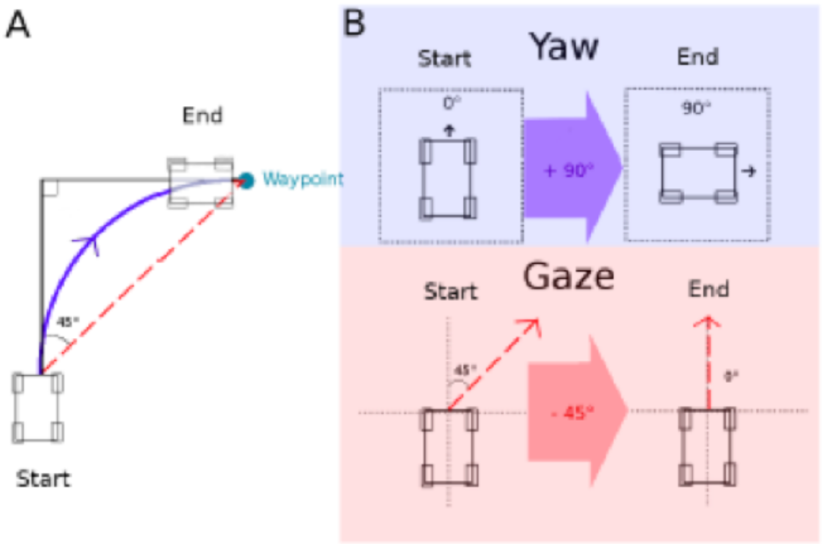

Supplementary Figure S1 Angular change of gaze when fixating a waypoint, as the observer rotates 90 degrees in the yaw plane. (A) visualizes a bird's eye view of car trajectory (blue) and the initial gaze vector (dashed red line) at the start of the turn negotiation manoeuver. (B) shows the change from the initial state (Start) and final state (End) for yaw angle – i.e. vehicle heading – in the world frame of reference, and gaze in the vehicle frame of reference.

Usually the point of fixation is not as far as in the example (i.e. horizontal gaze angles are below 45 degrees), but it is easy to see how the argument for a -½:1 ratio between the change in horizontal gaze angle and change in vehicle heading generalizes. I.e. Δgaze/Δyaw = -1/2 holds for tracking waypoints on a constant radius curve. When speed changes, but the trajectory remains the same, the time to achieve a given Δyaw will become shorter. Hence, the Δgaze/Δtime, i.e. horizontal angular velocity of gaze, must increase in direct proportion to speed and yaw rate.

Geometrical and dynamic considerations on the observer (or locomotor) frame of reference

When using the vehicle frame of reference as the observer frame of reference, as done in this study, some error will be caused because the above argument *strictly* holds for an

observer that does not experience yaw slip – i.e. observer body axis remains aligned with heading. In real vehicles this is not quite the case, because a. the observer i.e. driver is typically not located at the center of rotation, and b. the vehicle is not neutral steering but instead the longitudinal axis can point to the outside of the bend (negative yaw slip, understeering) or inside of the bend (positive yaw slip, oversteering).

For non-vehicle-assisted locomotion, such as running, the situation is even more complicated as the torso is not a rigid object, and does not have a unique longitudinal axis (e.g. shoulders and the pelvis rotate relative to one another, and current heading). Whereas the head is rigid and so an unambiguous longitudinal axis can be defined (although its direction will be somewhat arbitrary), gaze rotation in natural tasks is a combination of eye-in-head and head-relative-to-heading rotations – and it is the gaze relative to heading (not the longitudinal axis of any body part) that is relevant for gaze-locomotion coordination.

Thus, it is the heading frame of reference – not vehicle or body frame of reference – that gaze data should ideally be projected to. At the moment, however, this is unfortunately not technically feasible. The good correspondence with predicted values indicates that, especially for the simulator set-up, the effect of yaw slip should be negligible.

In a car, there will also be progressively more roll at higher speeds. This foreshortens the projection of the yaw plane (the plane of travel) in the vehicle chassis frame of reference horizontal plane (i.e. the plane that is orthogonal to gravity when the vehicle is stationary on level ground).

Supplementary methods

Participant instructions in the test track experiment

Clear and easily executed driving instructions were needed as we wanted a steady and slow rate of acceleration that would be comparable across subjects and between the test track and simulator experiment. The instructions were tested by piloting to be unambiguously understandable to an experienced driver. The instructions, translated from Finnish, given before the first run were as follows:

1.Drive the first round around the circle in first gear, with a minimal amount of gas and at a constant speed.
2. Drive the second round in first gear, accelerating slowly and steadily so that when entering the next round acceleration is smoothly continued moving into second gear
3. Drive the third round in second gear, so that the maximum limit speed is reached when entering the fourth round
4. Drive the fourth round at the maximum limit speed.

The “maximum limit speed” was explained to mean either that the participant's feeling of safety at its limit, or the vehicle reached its limit of grip and the car begins to drift and electronic stability control begins to activate. Participants were instructed always to drive so that they feel safe driving the car. They received no instructions where to look on the road

other than “drive as usual”, if they asked. No precise target speeds were given as glances to the rev counter or speedometer were not desired, although not expressly forbidden.

Signal post-processing

As the participants were unconstrained, there was eye movement in relation to the camera, camera movement (head and torso movement) in relation to car/screen frame of reference and car/simulated car movement in relation to the physical/simulated scene. Several stages of frame-of-reference transformation signal processing were thus necessary to connect eye-in-head data and yaw rate data (real and simulated).

Specifically, to correlate gaze and observer rotation in the horizontal plane, eye-in-head observations must be transformed into gaze-in-vehicle observations, and the horizontal component gaze velocity estimated in relation to the world. (In curve driving the head does a significant amount of tilting around the roll axis Zikowitz & Harris, 1999; Macdougall & Moore, 2005). In the results, gaze position is given with reference to the screen in the simulator and to the car in the test track experiment. (Thus measured gaze movement can be actually a combination of eye and head movement). The analyses were implemented by PR, with key algorithms developed by JP and additional code contributed by ST.

Head-in-vehicle positioning was done using optical markers in the camera view. In the test track experiment, optical markers securely attached to the vehicle frame were used to create a 3D model of the marker configuration fixed to the car’s frame of reference allowing head movement and tilt to be measured accurately in respect to the car’s vertical and longitudinal axes; in the simulator experiment markers were displayed directly on the screen image. From the 3D model, head position and gaze direction was calculated for each frame.

Camera distortion was corrected with cv2 undistort tool. Gaze was projected into the frame of reference of the car (test track) and the screen (simulator) on the basis of algorithmically identified optical markers in the scene camera images. Software for marker 3D localization (https://gitlab.com/jampekka/markerloc2) was developed by JP and additional software for simulator gaze projection analysis was contributed by ST.

The Pupil Labs software estimates gaze by identifying the pupils from the eye camera images. Custom code developed by ST to improve pupil identification in challenging (field) conditions (https://github.com/samtuhka/pupil). Bad calibrations were discarded and replaced with better calibration parameters from other calibrations.

Smooth pursuits were identified from raw (unfiltered) horizontal gaze position data using a de-noising and event detection method developed by JP called naive segmented linear regression (NSLR, Pekkanen et al., 2017; open source code available through links in the article). NSLR is a segmentation algorithm that approximates a maximum likelihood linear segmentation from the data points – i.e. between initiation and termination points of each segment gaze position is assumed to be changing approximately linearly. Angular horizontal gaze velocity was estimated for each (non-saccade, non-outlier) time point as the slope of the segment the time point belonged to. This is much more robust than using simple difference, and our algorithm does not require filtering of data prior to event detection, which can potentially change velocity characteristics.

For inclusion in the final dataset the segments were filtered so that only gaze fixations with pursuit type characteristics pass through (to reject spurious detection of saccades as pursuit). An absolute threshold for horizontal angular speed of the segment was set at 30 deg/s and accepted segment duration was set between 0.14 s and 1.5 s. (Note that these filtering parameters are not physiologically based: depending on task and stimuli smooth pursuit can be much faster than 30 deg/s, and natural task fixations shorter than 140 ms). The parameters were determined by inspecting the data and NSLR performance, to filter most of the saccades but still include most of the OKN-SPs. Maximum segment was not as really significant as the data didn't show pursuits over 1 second.

The Unix timestamps from each data source were used to combine the data from different sources. Yaw rate from the CAN-bus data was synchronized with gaze data via matching CAN-bus data velocity signal with the velocity-signals from the mobile phone synchronized with the eye tracker by TRUSAS. This made possible to link timestamps from the eye recorder-computer-phone system with timestamps from the vehicle CAN-bus.

In the test track experiment there was also the need to manually evaluate the start and end of each experimental run. When the car reached its limit speed, its Vehicle Stability Control System (VSC) started to kick in. Drivers needed to turn the wheel to compensate the movement fluctuation caused by shifts between a drift and the effect of the VSC. This caused drivers to jerk the steering wheel which resulted to quick changes in the yaw rate data. Compared to the steady cornering in the acceleration phase, this fast and intensive limit driving seemed qualitatively different. (Also worth noting is that drifting also changes the car's dynamics, especially roll, so that determining the horizontal reference plane of yaw-rate measurements becomes less and less reliable). Thus, it was decided to leave out from the analyses the final phase of limit handling, removing approximately 8 seconds of data from every run resulting in median of 84.0 seconds of driving per run.

Supplementary Results

Comparison the experienced subjects with the others

Simulator experiment data of the experienced driver participants who participated in both experiments (n = 4), were compared to those who drove only in the simulator experiment (n = 12).

Gaze velocity-yaw rate Pearson correlation coefficient for participants who participated in both experiments had a median value of -0.92 (mean -0.94, 95% CI [-0.97, -0.90]) and for participants who only took part in the simulator experiment a median value of -0.94 (mean -0.91, 95% CI [-0.97, -0.85]).

Orthogonal regression slope median for participants in both experiments was -0.90 (mean -0.93, 95% CI [-1.03, -0.84]). For participants who drove in the simulator experiment only the median regression slope was -0.97 (mean -0.97, 95% CI [-1.02, -0.91]).

As 95% confidence intervals overlap with both slope and correlation, these results do not suggest a statistically significant difference between these participant groups in correlation or regression slope.

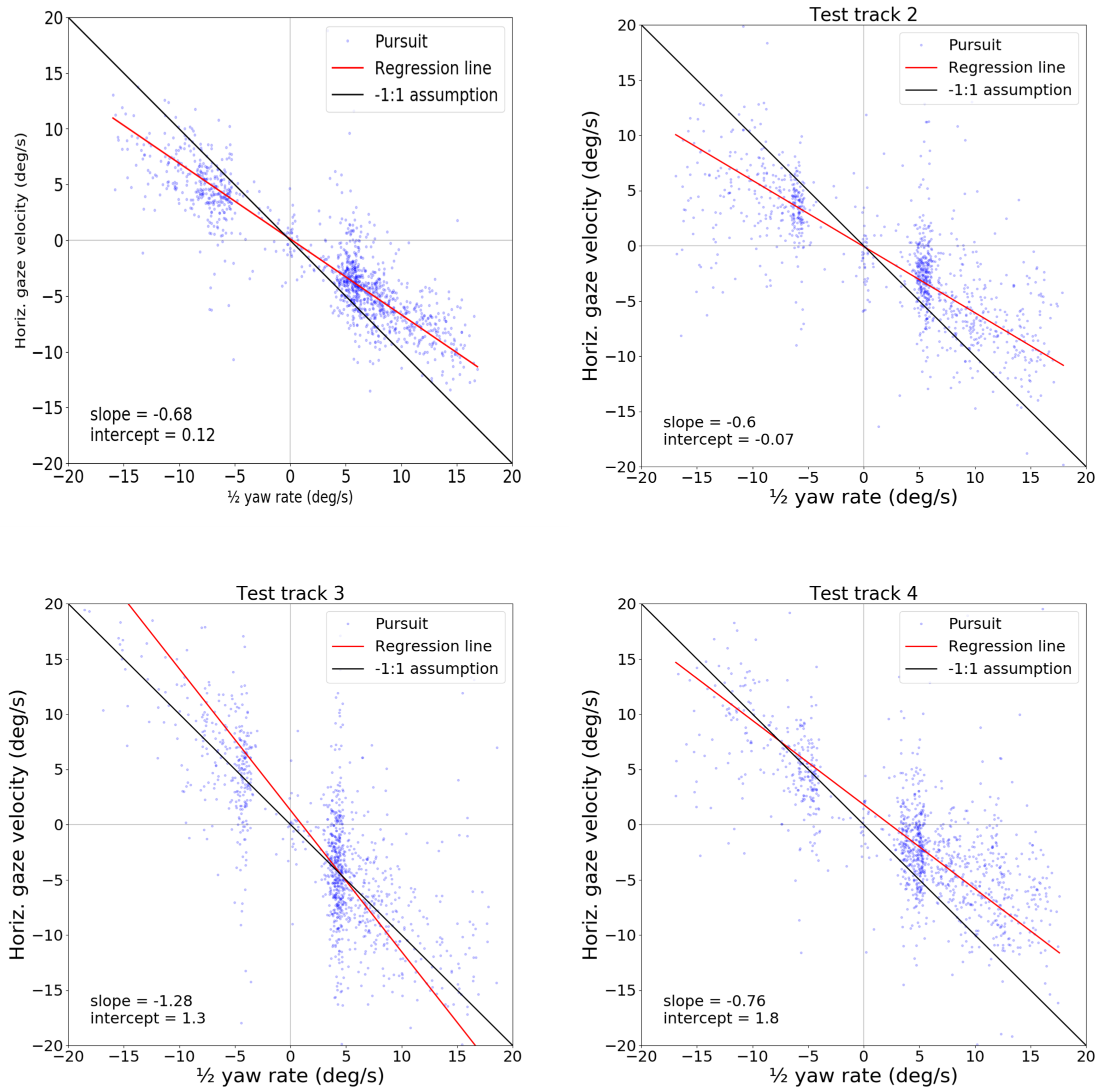

Supplementary Figure S2 Individual participant data of all four participants from the test track experiment. Blue dots are individual pursuits plotted against average yaw rate at the time of the pursuit. Red line is orthogonal regression fitted to the data. Black diagonal indicates -1:1 prediction.

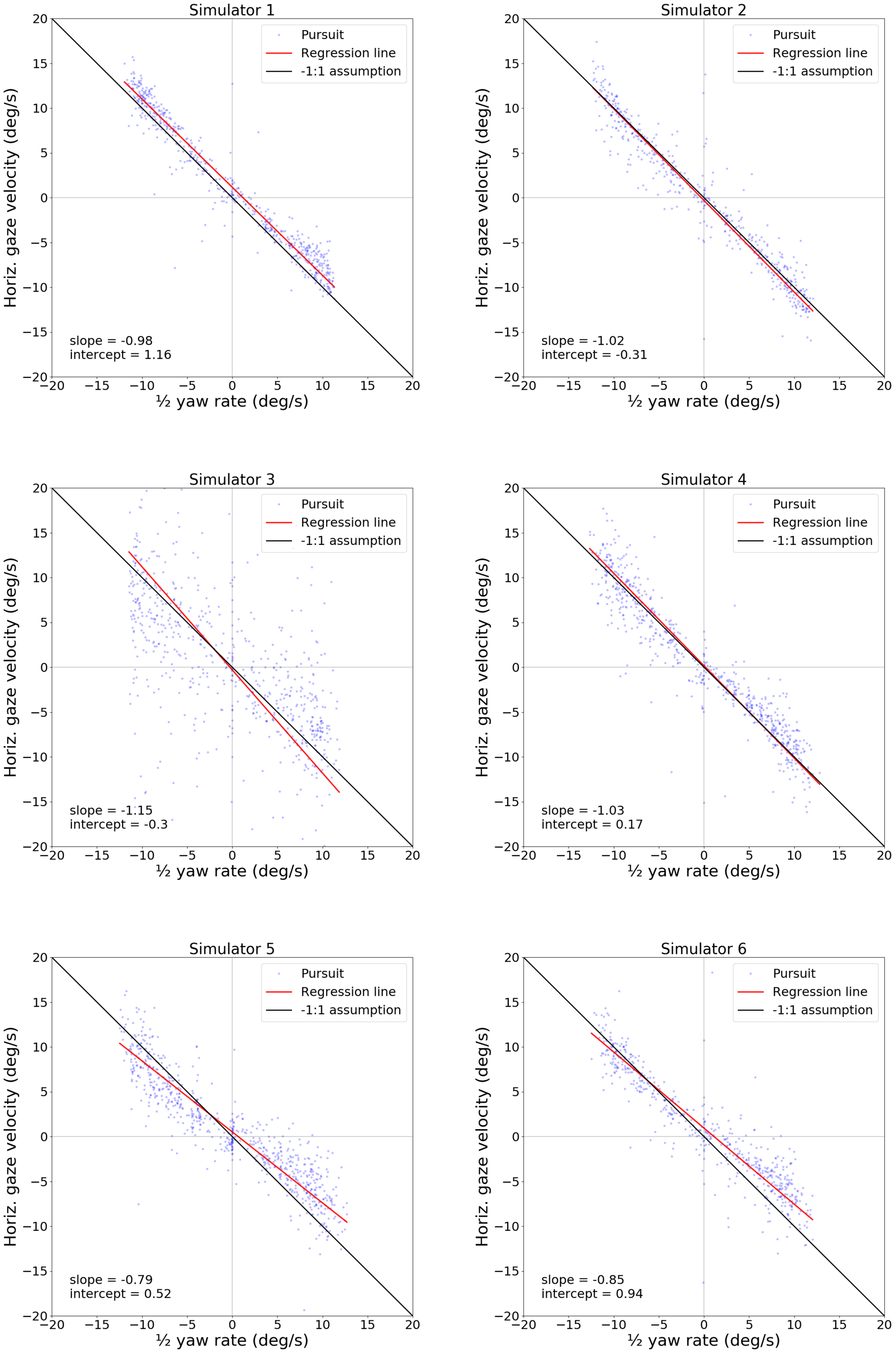

(*figure continues on the following page*)

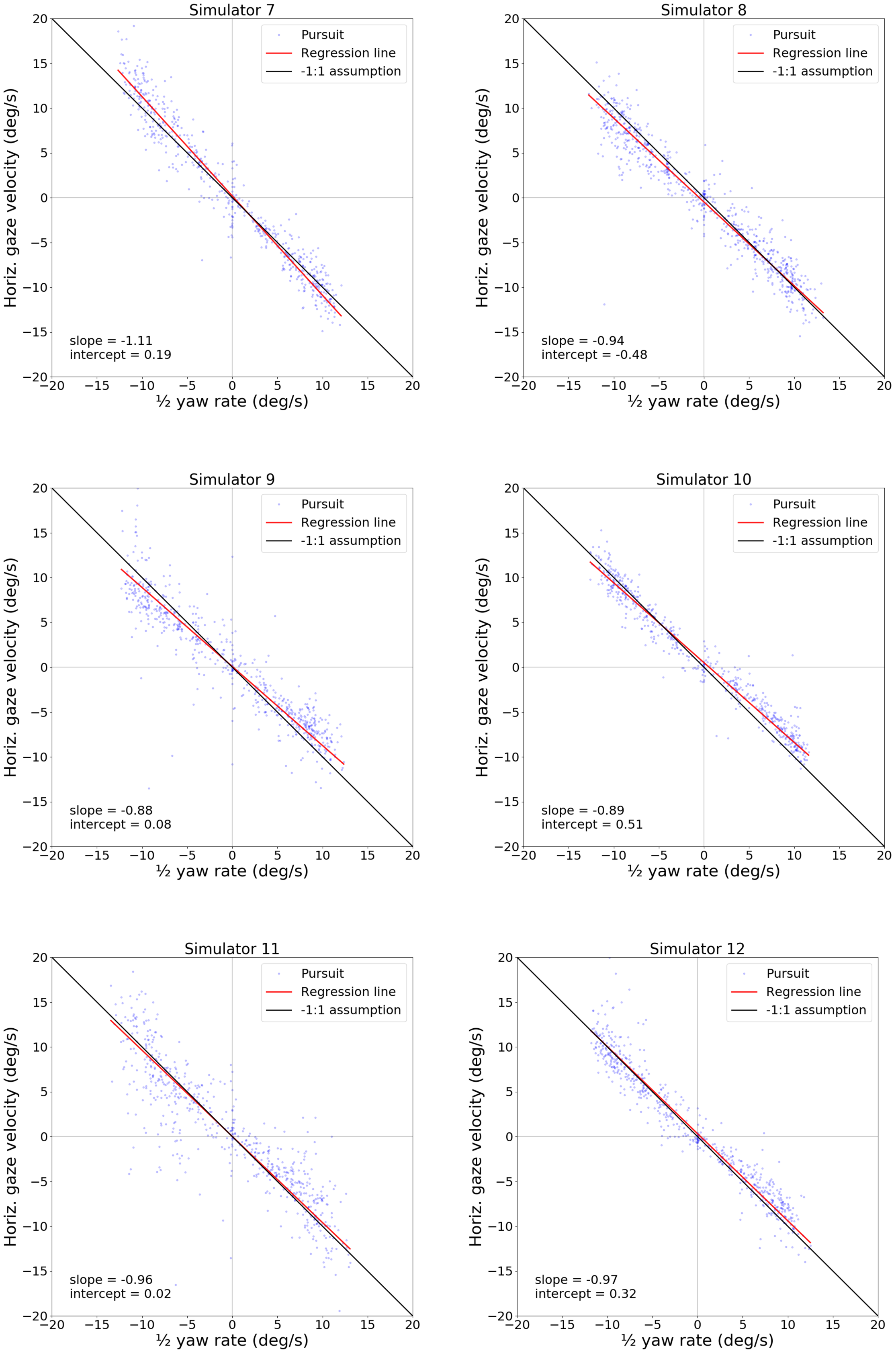

(*figure continues on the following page*)

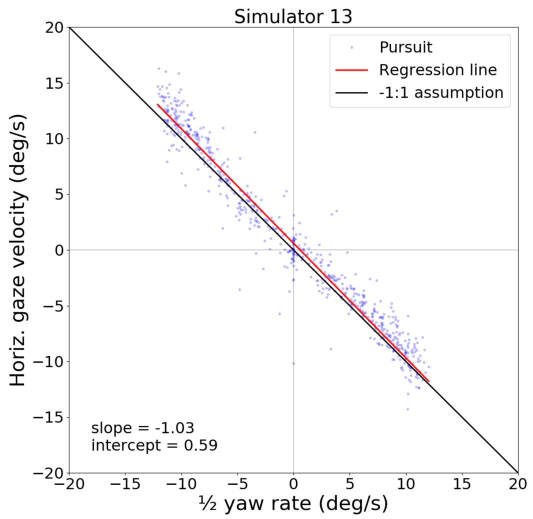

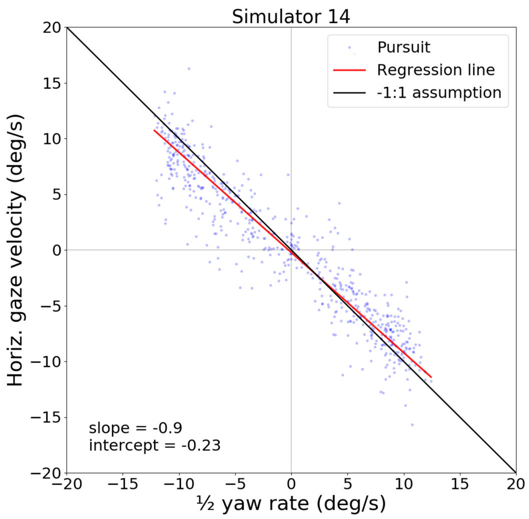

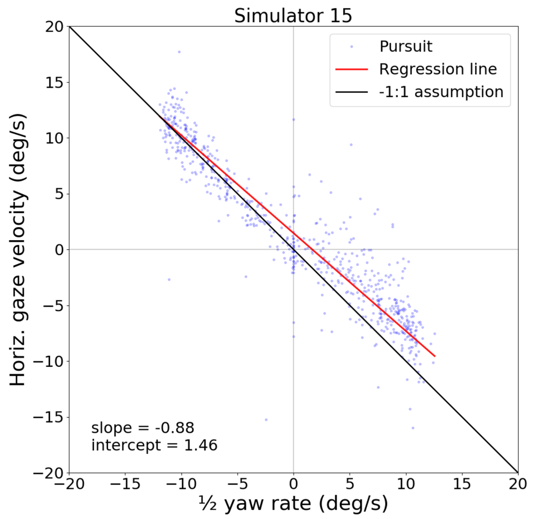

Supplementary Figure S3 Individual participant data of all fifteen participants from the test track experiment. Blue dots are individual pursuits plotted against average yaw rate at the time of the pursuit. Red line is orthogonal regression fitted to the data. Black diagonal indicates -1:1 prediction. Participants Simulator 13 - Simulator 15 are the ones who also took part in the track experiment.

REFERENCES

Zikowitz, D.C. & Harris, L.R. (1999). Head tilt during driving. *Ergonomics*, 42(5), 740 - 746.

MacDougall, H. G., & Moore, S. T. (2005). Functional Assessment of Head–Eye Coordination During Vehicle Operation. *Optometry and Vision Science*, 82(8), 706.

Pekkanen, J. & Lappi O. (2017). A New and General Approach to Signal Denoising and Eye Movement Classification Based on Segmented Linear Regression. *Scientific Reports*, 18,7(1):17726.
